# The basket trainer: A homemade laparoscopic trainer attainable to every resident

**DOI:** 10.4103/0972-9941.62525

**Published:** 2010

**Authors:** Nidal Jaber

**Affiliations:** Department of Urology, Dammam Medical Complex, Saudi Arabia

**Keywords:** Laparoscopic trainer, laparoscopic simulator

## Abstract

Laparoscopic trainers have been proved to be effective to improve skills of laparoscopic surgery; they are usually installed at hospital in the surgical department with limited access hours, usually inconvenient to the schedule of the resident. Simple trainer boxes are necessary for residents who desire developing their skills at home independently to the venue and hours of surgical departments. Our goal is to bring the laparoscopic trainer to the desktop of the surgical resident by making it very cheap, small, light, secure and easy to construct. We describe a model of laparoscopic trainer using steel basket which, we believe, meets all of the above-mentioned requirements. It is accessible to any personal budget and can be constructed with a minimum of hand skill. It is small and light enough to permit its daily use on the desktop of the resident for a couple of hours, then after it can be stocked in any locker.

## INTRODUCTION

Laparoscopic trainers were designed to help with training in basic laparoscopic skills and to assist surgeons in getting acquainted with instruments.[1,5,6] Numerous commercial virtual-reality and non-virtual trainers are available; however, since these trainers are costly they are unattainable by most surgical departments. Simple trainer boxes are necessary for the departments with limited budget and for residents who desire developing their skills at home independently to the venue and hours obliged by their departments. They have to be secure, small, and light enough to permit their displacement frequently and everywhere. The construction has to be easy, using cheap constituents and accessible in the markets anywhere in the world. They have to provide strong points of entry, while permitting a minimal freedom of movements. since the ideal angle of action and depth are not always easy to obtain in real practice of laparoscopic surgery, the ideal trainer has to provide a choice of several points of entry which permits a variable distance from entry to the target (10-25 cm) and a variable angle of action. A wide range of materials can be used to construct the box. Plywood, plaster of Paris, plastic box and desk drawer were previously used.[2–4,7] We describe a new trainer based on the use of a metallic basket. Our goal is to bring the laparoscopic trainer to the desktop of the surgical resident by making it very cheap, small, light, secure and easy to construct.

## METHODS

The parts that are required to build this trainer cost roughly only $41. As we will show in this section, nearly everyone should be able to construct it.

Other than the constituents in Table 1, a drill and a screw driver are necessary for the construction.

**Table 1:** Constituents of the model

Constituent	Additional description	Weight (grams)	Cost (US$)
A metallic basket	33 × 52 × 29 cm epoxy-bonded steel basket	800	14
Acrylic sheet	35 × 55 cm 2 mm thick	400	6
School ruler	3 × 30 cm	negligible	0.5
2 Hinges	With suitable screws	negligible	1
Rubber sheet	Rectangular rubbery mouse pad 20X30 cm	negligible	1.5
Cork sheet	20 × 30 cm	negligible	1
Hook and loop strap	1 × 50 cm	negligible	1
Pins	4 pins for cork sheet	negligible	0.5
Camera	Cheep webcam with clamp	50	11
Adhesive tape	a roll of PVC electrical insulation tape	negligible	1
Glue	a tube of 20 ml of all purpose adhesive glue UHO® l	negligible	2
Total		1400	41

**Figure 1:**
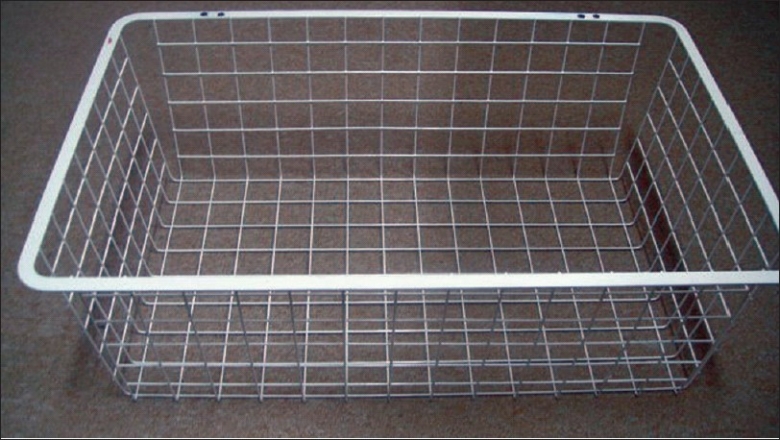
Metallic basket

**Figure 2:**
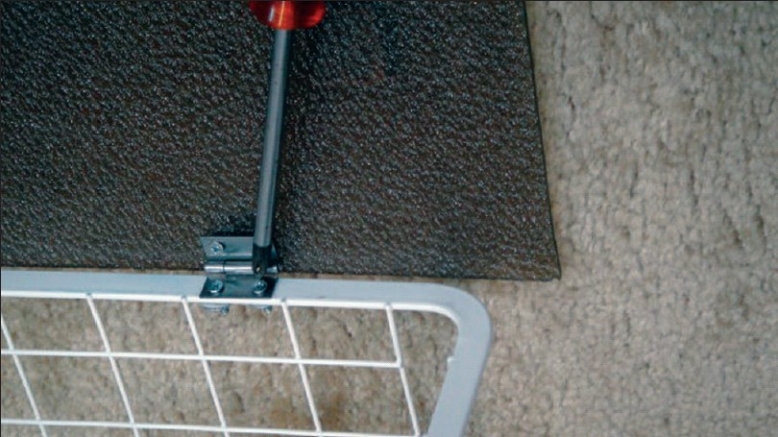
Installation of the hinges

**Figure 3:**
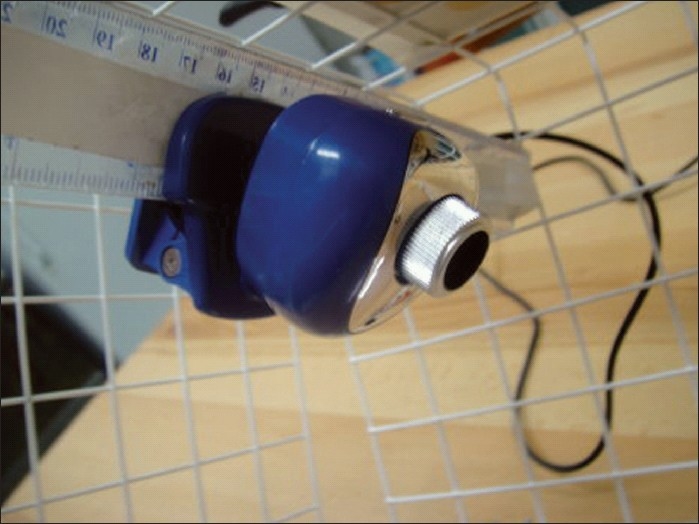
Attachment of the camera

**Figure 4:**
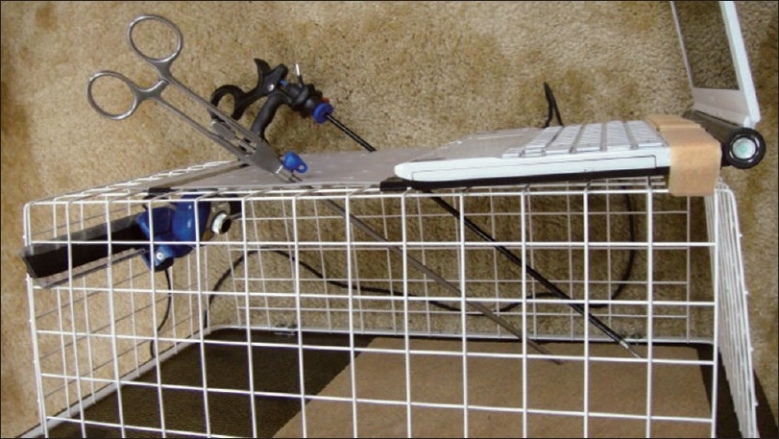
The Trainer is ready to use

1. **Assembling the box:** The basket we use for our trainer is a runner drawer designed by Elfa® for customized shelving and drawer system. It is robust and light, made of strong epoxy-bonded steel and available in showrooms and through internet (cost: 14 US$) [Figure 1]. An acrylic sheet is used as a base for the box (polycarbonate and plywood sheets may also be used). Use a drill and screw driver to install the two hinges on both the basket and the acrylic sheet on one side only [Figure 2]. No lock is necessary for the opposite side since the basket will drop down over the acrylic sheet by its weight.
2. **Attaching the camera:** Any web camera in the market is suitable given it can be attached by a clamp to the ruler previously introduced through the eyes of the basket in a high and posterior position and fixed by tape [Figure 3]. In our early experience we used a web camera with incorporated light (cost: 13 USD), but we quickly realized that we do not need any light source since the basket permits the passage of enough light for excellent vision. Optional upgrading, naturally more costly, may include high definition camera, digital zoom, auto focus, motorized tracking or a colour surveillance camera connected to a screen.
3. **Preparing the points of entry:** A rubber sheet (or a rubbery mouse pad) is attached over the top of the basket by tape. By its position, it will prevent the direct vision of the target. It will also permit by its elasticity the degree of freedom necessary for the point of entry [Figure 4].
4. **Preparing the working area:** A cork sheet is fixed to the acrylic sheet by glue UHO®. The piece of tissue to be sutured (or anyother convenient material) may be attached to the cork sheet by pins.
  **No computer** is included. Every trainee will pose his personal laptop over the basket and fix it by a Velcro® hook and loop tape [Figure 4]
5. **Instructions for use**
  1. Connect your laptop to the camera.
  2. Install the driver using the provided CD (This is unnecessary for plug and play cameras)
  3. Adjust the direction, focus and zoom of the camera.
  4. Put the laptop over the box and fix it by Velcro® hook and loop Strap.
  5. Choose the suitable points of entry on the mouse pad and make the desired holes by knife or trocars.
  6. Introduce your instruments and start training.

## DISCUSSION AND CONCLUSION

A new simple laparoscopic trainer is described. It is cheap and easy to construct for most of surgery residents, small and light enough to permit its daily use on the desktop at home. This basket trainer will permit to overcome the difficulties due to the unavailability of trainers in the hospital or their limited access hours by bringing the laparoscopic trainer to the desktop of the surgical resident.

The characteristics of our model compared to previously described trainers are as follows:

- Time to construction: 30 min.
- No need for hand skill. It is mainly a simple assembly,
- All the constituents are readily available in the market are ready to use.
- The epoxy-bonded steel basket is strong and light, it permits a free passage of light and air making the use of light source unnecessary and providing an excellent security with no risk of overheating or electrical shortcut.
- No direct vision is possible since the rubber sheet stays in the axe of vision.
- Overall weight of the trainer (without laptop) is 1400 g.
- Dimensions: 55 × 35 × 29 cm.
- Overall cost: 41 US$ (laptop not included).
